# Footfalls on the Boundary of Another World

**Published:** 1860-07

**Authors:** 


ART. III.—*Footfalls on the Boundary of Another World. With Narrative Illustrations.* BY ROBERT DALE OWEN, formerly Member of Congress, and American Minister to Naples. 12mo., pp. 528. Philadelphia: J. B. Lippincott & Co., 1860.

WE can scarcely, we think, be accused of transgressing the proper province of a medical journal by admitting into it the review of a work of the character of that before us. Whether the phenomena discussed by the author be admitted to fall within the sphere of the normal functions of man's intellectual or spiritual nature, or shall prove to be the results of certain diseased conditions of the material organs through which the operations of the mind are performed during man's present state of existence, they are in either case legitimate subjects for medical investigation.

The questions discussed by Mr. Owen are equally curious, solemn, and momentous. The very statement of them is sufficient to excite in the mind of all a feeling of the deepest interest. What relationship does man hold during life, with the disembodied spirits of those who have already passed from the present stage of existence? Is man subject to ultramundane influences, and what is the nature and extent of such influences? Can the immaterial portion of man's nature act, before death, independently of material organs, apart and at a distance from the body?

To neither of these questions has the author attempted to frame a definite answer. The entire course of his reasoning, nevertheless, indicates a strong inclination on his part in favor of the affirmative in respect to all of them. To establish the correctness of this position is evidently the tendency, in his estimation, of the several narratives of spiritual manifestation and interposition he presents: to the same end are directed the commentaries with which these narratives are accompanied.

With an assumption of the most perfect candor — disclaiming the slightest intention to undertake the support of any preconceived theory in relation to the subject, but professing to present simply a series of the best authenticated narratives bearing directly upon it, leaving each one to judge of these according to the claims they are found, after the closest scrutiny, to present to the character of credible facts, and to draw from them just such conclusions as it is believed they legitimately lead to—Mr. Owen, in the work before us, has prepared one of the most ingenious and seductive defenses of the reality of ultramundane visitations and influences—of suggested dreams and visions of a prophetic, monitory or advisatory character, and of the ability of the spirit that is in man to act independently of the material body with which, for all the ordinary purposes of life, it is conjoined. He has selected and arranged his evidence with consummate skill; while the commentary by which the different narratives adduced by him are followed and enforced is of a character the best adapted to compel his readers to the very conclusions at which he desires they should arrive.

With the nature and mode of being of man's purely intellectual or spiritual portion, and the manner and extent of its connection with the animal organism we have, it is very certain, but a limited and imperfect acquaintance. We are far from having made ourselves masters of all the powers and faculties of man's mind, or soul, or spirit, his immaterial portion, call it by whatever name we may. We know not but that it may possess faculties, and perceptions, and modes of being and of acting that have as yet been revealed to us but in part. It may be able, for aught we have the right to assert to the contrary, to perform many functions altogether independent of the material portion of the animal organism—to receive impressions from ultramundane influences by other routes than those of the senses—to see and hold communion with disembodied spirits—to pass out for a season from its clayey tabernacle and assume a visible form capable of moving, speaking, and acting without the intervention of material organs—to be uninfluenced in its perceptions or actions by time or space, so as to know what transpires in the most distant places, and to look forward upon the events of the future long before they happen.

It is not our province to stamp with the seal of falsehood any narrative which is presented in proof that the spirit of man does really possess the powers and faculties above enumerated, in apparent good faith, by those whose veracity is unimpeached, and in whom we can detect no motive sufficiently powerful to induce them to deceive themselves or others. We have no authority to reject as unworthy of credence anything adduced as fact merely because it is not in accordance with our experience or does not accord with our conceptions of what is true. It is our duty, however, to examine into the probability of the evidence which is urged upon our acceptance in support of any and every proposition, and into the competency of those from whom it is derived.

An individual may tell us—and really believe that he is telling us the truth and nothing but the truth—that he saw certain objects, was present amid certain scenes, had heard certain voices or sounds, or experienced certain impressions, and yet from many causes may be an incompetent witness, either from the character of his mind generally, or its particular state at the time being,—from some predominant idiosyncrasy or special abnormality of his sensations and observing faculties generally,—or from the simple fact that his powers of observation are undeveloped or not properly educated, and, in consequence, loose, superficial, and inexact, if not positively false. The simple qualification of veracity, combined even with the very best intentions, is not in every instance all that is necessary in order that the statements of an individual shall be received as the certain exponents of absolute facts. To admit him as an unquestioned evidence, we must be sure that his observing and judging faculties have been properly cultivated; that they are without any undue bias from education, superstition, or fear, and have not been rendered unapt to appreciate the actual and the true through some defect in the organs through which images and sensations from objects without are transmitted to the mind.

We know that it is possible for truthful, honest, well-meaning persons to see men as trees walking, to hear strange words whispered in the sighing of the breeze upon the ocean strand, or groans and cries of anguish, "as of suffering or grieved spirits," in the natural sounds that occur, amid the recesses of some unfrequented forest; or when awed by the unreal imagery by which the hours of sleep are often haunted to have revealed before their mental sight, visions, as they believe, of things intended to be done by others—of things about to happen to themselves, or of events that have already transpired or are about to transpire in their own vicinity or at a distance.

The senses of some men always play them false—deceiving them continually in respect to the forms, and colors, the nature, conditions, and relations of the objects upon which they look. Many mould all things that meet their vision after the ideal patterns that they have formed and treasured within their minds; while others look with open eyes upon the objects that are before and around them, without in truth observing them, so that when they turn aside from viewing them, as these have failed to make any distinct and definite image upon the mind, they forget entirely what manner of things they were.

In reference to the most common every-day occurrences which fall within the appreciation of every one, how difficult is it to obtain correct and reliable accounts! Now, how much greater must be the difficulty in reference to things of an unusual, obscure, and startling character—calculated to excite in the coolest and most courageous beholder deep fear or the profoundest awe! If, then, as we all know does occur frequently, the accounts given of the same transaction by different spectators will differ from each other in the most essential particulars, even when the transaction is one of the most simple and familiar character, and all the spectators who testify of it are equally qualified and competent to observe and judge correctly, how are we to expect accurate, clear, and reliable accounts of occurrences of a most extraordinary character, seen always under circumstances the most unfavorable for deliberate and accurate observation, and by those who are themselves often predisposed to be led astray in respect to everything that differs materially from the known and the familiar?

All the external senses are constantly liable to perversion, either from some congenital defect of their organs, or as the result of some deep-seated, obscure disease. This is especially true of the sense of sight, which in different individuals—without any visible defect of the eye itself—has been found to be incapable of distinguishing aright the colors, forms, proportions, and relations of objects; mistaking often shadow for substance, or seeing in the open air, and in the full light of day, objects variously distorted, or images, distinct and clear and prominent to others, as though they were enveloped with a thin mist or by clouds or smoke. And yet, with all these sources of deception, we are called upon not only to receive as absolutely correct the accounts that are given of a variety of phenomena which are said to have occurred out of the ordinary course of things, but to admit as correct the explanation, also, of the relators in respect to their nature and cause.

Such are some of the difficulties which encompass, to a certain extent, most of the narratives of things, phenomena, and events claimed as pertaining to the spirit or soul of man when acting independently of his bodily organs, either previous to or after death. They are sufficient to throw around such narratives a reasonable doubt as to their entire accuracy, though it is admitted that they are alone insufficient to invalidate them entirely. But if, in connection with the defect alluded to in the evidence upon which the narratives are based, they are justly amenable, also, to the charge of improbability, then we are bound to reject them until the difficulties in the way of their reception as truths be satisfactorily removed. How far the evidence in support of all or any of the narratives collected by Mr. Owen may be viewed as defective, and to what extent they may be pronounced improbable—not because of their unusual or mysterious character, but from the inconsistencies apparent on their very face—we leave to the decision of all who may be inclined to submit them to a severe criticism, the test of which many of them, we fear, would scarcely stand.

In regard to all supposed supernatural occurrences, we have a right to demand fuller and more detailed accounts than those with which we are usually furnished. To their satisfactory solution, it is essential that we should be put in possession of many preceding and contemporary facts which, if not withheld entirely, are in nearly every case related with great looseness, and in a manner which proves that they have not been subjected to any systematic or close investigation. We are thus deprived, in most instances, of the only means by which it is possible to solve the mystery in which many of the narratives adduced as evidence of spiritual agency would seem to be involved.

An argument in support of the truth of ultramundane influences and various forms of purely spiritual agency in the affairs of men is drawn from the universal belief in them which has existed among all peoples, and, so far as our knowledge extends, in every age: so that hosts of stories of ghosts, apparitions, and visions, confessedly of the most questionable authority, have been received by the masses, without the slightest doubt being entertained as to their authenticity. Now it is argued that if there had never actually occurred an instance of ultramundane influence or spiritual agency in respect to the individuals of our race and their affairs, no credit would ever have been awarded to the tales invented in illustration of such occurrences. The argument, however, is of little force. It is not necessary that the spirit, unclothed with flesh and blood, or airy shapes from the realm of death, should have presented themselves before the natural eyes of man when wide awake, or to his mental vision when his external senses are "fast locked up in sleep," in order to create a belief in spiritual appearances and influences. The rise of that belief has not been in the real, the actual, the positive, but rather in the imaginary, the unreal, the visionary. The originals after which all the tales of ghosts and spectres—the warning and prophetic appearance of departed or absent friends or enemies, admonitory or suggestive dreams, ultramundane communications; the entire stock, in short, of ghostly literature and tradition—have been modeled after, are to be sought for, in fact, in the ocular spectra, which, under various circumstances, form themselves before the eyes of men or upon the mind, when the visual organs are shrouded in sleep, and with so exact and vivid a semblance of reality, as to leave scarcely a doubt that they had been transmitted to the mind through the sight from corresponding objects having a positive existence without. As such they have been received, not merely by persons of exalted imaginations, strong prejudices, and slight intelligence, but by those also of unimaginative, practical minds, unquestioned veracity, and cultivated intellects. But, it may be asked, are we warranted in ranking every instance adduced as one of a purely spiritual character with these chimera—the offspring of disordered vision, or of a partial and disturbed action of the mental functions? We have no intention to reply to the question with a positive affirmation or negation; all that we insist on is, that every reputed instance of the kind shall be subjected to that close and cautious investigation necessary to determine its real character—whether it in fact have its origin in ocular spectra, in mental illusion, or in spiritual agencies and influences altogether independent of natural organs.

There is an all-important question to be solved—one standing, indeed, at the very threshold of the investigation to which we have been invited by Mr. Owen in the work before us, and without a satisfactory solution of which every step we attempt to make in search of the true doctrine in respect to the spiritual influence and agency, said to be exercised between persons who still live upon the earth, must be in the dark; and that question is, can, under any circumstances, the immaterial, the spiritual portion of man, his soul or intellect, appear in a visible shape, separate from the material body, during his continuance in this world? Is it possible for it, excepting through the instrumentality of material organs or means, to act upon persons near or afar off, so as to reveal itself to the latter, to communicate with them intelligibly, or to influence them in any manner whatever?

Judging from all the facts to be derived from the domains of physiology and pathology, from our personal experience, and that of the great majority of those who have written or spoken on the subject, we shall have to answer the question referred to in the negative. So intimate is the connection between man's moral, intellectual, or spiritual nature and certain of his bodily organs, that we find its development to keep pace with the development of the latter, and to receive always from them a sensible modification according to their condition as to health or disease; nay, to become entirely suspended in all its manifestations—extinguished, as it were, when the organs referred to are the seat of certain lesions. Pitcairn tells of a beggar, whose brain had been laid bare in consequence of the removal of a portion of the skull, whom the students of the medical school at Edinburgh used to experiment upon by subjecting the brain to pressure, gradually increased to an extent compatible with the entire safety of the patient. The invariable effect of pressure applied to the brain in this case, was to throw the individual into a condition of complete unconsciousness, which continued as long as the pressure was kept up, and ceased as soon as it was discontinued. A friend of ours while riding was thrown from the back of his horse, and experienced in consequence a severe concussion of the brain. He remained for nearly five days in a state of unconsiousness. After he had entirely recovered the exercise of all his faculties, he assured us that, during the whole period intervening between his fall and his return to consciousness, he felt nothing, saw nothing, heard nothing, knew nothing; that he was as though he had no being. On coming out from this state, as it were, of deep dreamless sleep, he connected the time when he awoke to consciousness with the moment of his accident—the whole of the intervening time had, as far as he was concerned, been completely annihilated. Many cases precisely similar are on record. Now, if man's intellectual powers, which we presume will be generally acknowledged as intimately connected with his spiritual or immaterial nature, are so closely joined with his material organs as to suffer from every disease and derangement of the latter, is it reasonable to suppose that the spirit possesses the power to disengage itself from the material organism, and perform some special mission apart and independent, it may be at a long distance from the latter, either without the volition of him to whom the spirit appertains, when prompted by intense desire or some strong moral impulse, or else in obedience to the dictates of a determined and irresistible will?

History and biography furnish us with numerous instances in which individuals of determined purpose and iron will, worthy people too, and of good report, have been so circumstanced that, for the vindication of their character, for the preservation of their lives, and for the safety of those near and dear to them, there was a pressing necessity they should communicate with certain persons, but were prevented from so doing by physical force. Now here are precisely the instances where we should presume that the spirit, if it have the power, would assert its independence of the body, and perform the much-desired errand of mercy. But history and biography commemorate no such good performed by the spirit as a disembodied spirit.

By all believers in the power of man's spirit to emancipate itself during life from the body, and to act independently of it, an effort is made to prove that it is continually active; that under all the circumstances of life it is so far independent of the animal organs that, during the physiological state of repose into which these periodically fall, it slumbers not. That, consequently, the mind being always active during sleep, sleep is never dreamless, although it is not always that the events, thoughts, and emotions of dreamland are remembered by the individual upon awaking. Even were this position susceptible of proof, it would be very far from establishing the independent and unceasing action of the intellectual portion—the spirit, soul, or mind of man. Dreams, properly speaking, it has been very clearly shown, never occur during that state of complete quiescence in which all the organs, save those which are removed from out the dominion of the will, periodically sink, in order that by repose their powers and energies may recover from the exhaustion incident to the active exercise of their functions during the waking state. Dreams consist in nothing more or less than disjointed images and broken chains of thought revived in the mind of the sleeper, in consequence of certain of the cerebral organs having regained a greater or less degree of activity, during a period when external consciousness and the judgment remain dormant. They are invariably founded upon images and thoughts pre-existing in the mind of the sleeper, but when thus revived, during a state of partial or imperfect sleep, they become grouped, commingled, and distorted into a medley of inconsistencies and contradictions, as is apparent to every one upon a careful analysis, after waking, of the most coherent and vivid of his dreams.

Dreams cannot be adduced as examples of intellectual or spiritual action independent of the material body. They consist evidently of a series of phantasms beheld by the mental vision, exciting often fear or disgust, admiration or joy, approbation or condemnation.

"In dreams," very aptly remarks the venerable Parr, "we seem to reason, to argue, to compose; and in all these circumstances, while the dream lasts, we are highly gratified, and think that we excel. If, however, we remember our dreams, our reasoning we find to be weak, our arguments false and inconclusive, and our compositions trifling and absurd. Some metaphysicians have supposed that from age and reflection our dreams become more consistent and philosophical, and have even supposed that the mind during sleep retains its wonted powers or even augmented powers. We are willing to believe that, from age, our minds wander less when dreaming, but we suspect this arises from the sleep being then less perfect, and not from any experience acquired in 'the art of dreaming.' We certainly fancy in our deams that a given image is new, but if we can retain it when awake, we find this opinion arose from our imperfect recognition, and we shall then, perhaps, be able to recollect its prototype. We seem to think, also, some place, which in fancy we see in our sleep, to be more beautiful and glorious than any which has before occurred. Yet on awaking we shall find this splendor a thing of shreds and patch-work, made up of heterogeneous and disjointed vestiges before offered to the senses."

It will be urged, no doubt, that during the state of dreaming musical pieces have been composed, poetry has been written, and the most intricate problems in mathematics have been solved, and with a facility and in a style far exceeding anything of a similar kind before attempted by the same individuals during their waking hours; and it will be insisted on that this can be accounted for in no other way than by attributing these splendid efforts of the dream state to an increase of power and of clearness in the intellectual powers of man, when these are emancipated from the dominion of the material organs in consequence of the inactive condition into which the latter are cast by sleep.

We admit that the most brilliant and extraordinary mental achievements have been executed by persons who were at the time in a state of supposed sleep; but we believe that the supposition has in every instance been founded in error. So far from the individuals by whom the intellectual feats to which we have alluded were performed being immersed at the time in sleep, sleep, in the proper acceptation of the term, was afar from them. Their condition, we apprehend, was one of complete mental abstraction: their minds were withdrawn entirely from, and their senses closed, as it were, upon all the material existences by which they were surrounded, and the full force of their intellectual powers, with naught to distract or turn them aside, were concentrated on a single subject. Working thus, with a degree of energy that can never be equaled out of the state of abstraction to which we refer, it is not at all surprising that the mind should then execute tasks in a manner which at all other times is found to exceed its ability.

A very pertinent query suggests itself, and that is as to the condition of those whose spirits are supposed to have gone forth on missions of mercy or of retribution, of prophecy or of warning, during the period when the spirit is absent from the body. Take for instance the female teacher of whom Mr. Owen tells us, whose spirit is said to have occupied her usual seat in the school-room on occasions when her body was walking and culling flowers in the garden, or the man whose spirit we are told left his body on board a ship in imminent danger of being crushed by an iceberg, while it went afar over the bosom of the ocean in quest of relief for the ship's crew. We would inquire, and we insist that the inquiry is a proper and reasonable one, what was the condition of the body in these instances after the spirit had left it, in respect to mental or intellectual manifestations? Was it in a conscious or unconscious state? Were its actions, supposing it were capable of any, merely automatic, without the guidance of reason, without object or purpose? It certainly will not be asserted that an individual is able to act, and converse and reason as well when his spirit has gone out of his body on a visit to some other spirit, in or out of its proper body, as when it is at home within its own earthly domicile.

The ability of man's immaterial intellectual portion to act independently of and at a distance from his material portion, depends, it is assumed, upon the fact that man possesses two distinct bodies, a spiritual one and a material one, the one being to a certain extent the similitude of the other. Now in all the accounts that are given of the appearance of the spiritual, distinct from the natural body, it not only bears a general likeness in form, carriage, and lineaments to the person to whom it appertains, but it is described, usually, as being clothed in garments similar to those which the latter, or persons of his or her age and condition in life, are known to wear. Are we, then, to infer that the garments men wear have also two bodies—that all, whether it be the full armor of the warrior of the olden times, the full-bottomed wig, broad skirts, slashed sleeves, and ample costume of the ancient gentleman, or the more familiar attire of our contemporaries, to say nothing of female dresses, from the times of our remotest ancestors to the present, have alike a material and a spiritual portion, capable of separation, the one from the other? If such be not the case, what then, we ask, is the nature of the raiment with which the spirit, when apart from the body, is invested? Is it material or immaterial? It has been described as having, in certain cases, had the look of some particular stuff used for clothing, and as giving some indication of substance to those who attempted to grasp it, or against whom it accidently brushed in passing. Is it supposed that the spirit, when it is about to leave for a season the body of some unconscious sleeper, comes forth with the similitude of the garments usually worn by the latter, or does it appropriate to its use some immaterial pattern of these after it has become disembodied? It will not do to insist upon the actuality of supposed spiritual appearances, and at the same time to admit that the clothes with which they are clad are mere ocular spectra. Upon the doctrine that this latter is the true character of all these appearances, everything in relation to them, whether it be clothing, deportment, or facial expression, will be found to have an immediate and appropriate connection with the circumstances under which the spectra are brought before the mental or material vision.

We have a firm belief in the complex nature of man. We recognize him as a being composed of a material organization, changeful and perishable, and an immaterial mind, soul, or spirit, ever existing, immortal. But we believe that these two natures are, in this state of existence, intimately knit and bound together, so that they alternately influence and are influenced by each other, and that it is only after the dissolution of the first that the latter is capable of enjoying an independent existence. It appears to us, from all that can be learned of the physiological and psychological history of the human being, that so long as his earthly career endures, his immaterial portion, his mind, soul, or spirit, acts and is acted upon only through material organs.

We controvert not the declaration of St. Paul, that "there is a *natural body*," and that "there is also a *spiritual body*," for every individual of the human family. But, with St. Paul, we believe that no one has been or can be invested, under any circumstance whatever, with "the spiritual body," until by death he becomes freed from "the natural body."

The question now presents itself, has any one, after he has become, by the death of the material body, invested with that which is spiritual, ever appeared to those who are still in their natural bodies? The possibility of such an occurrence we have no right to question. We can see nothing improbable in the belief that, for the accomplishment of some important mission, having in view the moral good of a human being, the departed spirit, say of a parent, husband, wife or child, or of some loving and beloved friend, may be prompted, and permitted to exert upon him or her its influence either secretly, or through some visible manifestation to the external senses or in the similitude of a dream. In this we can see nothing improbable. With our present views of the nature and probable occupation of the spirits of the departed, we cannot, however, believe that they are ever permitted to intermeddle with the affairs of mortals on the most trivial occasions, as some of the stories told us would lead us to suppose, or even, on occasions, with no possible object in view, certainly with no results sufficient, as would appear to us, to call for spiritual influence to secure their accomplishment. Far less can we suppose it possible that disembodied spirits would come from the state beyond the grave to commit any of the many follies that have been ascribed to their agency—to disturb the peace and quiet of decent and well-governed families by the production of mysterious and discordant sounds—by causing furniture to be thrown about in wild disorder, or by inflicting secret blows upon the domestics or other inmates of a house.

If the spirits of the departed ever do come to minister to us from beyond the grave, or to bring to us a message for our good—to admonish us of sin, to warn us of impending evil, or to snatch us from the machinations of some secret enemy, we feel persuaded that their coming will be invariably in such a form and manner as to give solemnity and weight to their mission—the object of which will be accomplished by them with a directness and clearness calculated to leave no doubt or mistake or misgiving in the minds of those who are its immediate objects. Spirits freed from earthly organs—pure intelligences, acting as independent essences, unimpeded and untrammeled by things material, cannot be suspected of performing their errands toward man—if such errands be ever performed—in so indirect and bungling a manner as do the imaginary spirits of which we read in books—of paltering with those with whom they hold communion in phrases of double meaning, like the oracles of old—or of dealing in insinuations and suggestions of only dark and equivocal import, in place of speaking out in simple, unequivocal language whatever they have to communicate, whatever they desire of those they visit.

We are pointed to the histories which have been given to us, upon the authority of intelligent and veracious individuals, as proof that the spirits of the dead have appeared to mortal men, and on appearing have conducted themselves in the very manner in which we contend they would not have done, had they been truly visitors from spiritland, and that too, in the presence not of a single witness, but of many—not alone, in the silent watches of the night, nor in the dim twilight, but in the open fields, the thronged thoroughfare, and the light of day. Not once only, but repeatedly, and in a manner to preclude mistake.

We are well aware that all this is told, and, in many instances, it may be, in perfectly good faith, by witnesses esteemed in every respect competent. We do not deny that many of the appearances and occurrences—the sights and sounds asserted to be of a spiritual character, were actually seen and heard by those who relate them. The question with us is not, were the things described witnessed, but rather, what was the true character of the things that were seen or the sounds that were heard? In classing all supposed spiritual appearances and occurrences as ocular or mental delusions, we by no means restrict their appearance to one individual at a time, although such is the fact, we believe, in respect to most of them. There are unquestionably many natural phenomena which may appear, at one and the same time, to a number of spectators, and make upon all of them the same impression, and nevertheless be nothing more than optical delusions—forms, often without substance, or, perhaps, substantial objects, of which the forms and general aspect have, for the time being, been essentially modified—or the objects themselves apparently displaced and disarranged, by some peculiar accidental arrangement of light and shadow, or by some temporary change in the dioptric properties of the atmosphere.

We are far from pretending to be able to give to all the accounts that have been published of spiritual interposition a clear and satisfactory natural explanation. We want the necessary data to enable us to arrive at any certain conclusion in regard to the true character of very many of them. Were we in every case put in possession of all that is requisite for the formation of a correct judgment, we have no doubt that the non-spiritual character of the whole of them could be easily shown. Still, the glaring contradictions, and the general improbability by which the more prominent of the narratives are characterized, are circumstances sufficient of themselves to warrant us in the rejection of them from the class of spiritual occurrences, even while we find ourselves at a loss in our attempts to develop their actual nature.

In confessing, as we have done, that there is no improbability in the supposition that the spirits of the dead are permitted, for some good and wise purpose, to exercise an influence over the inhabitants of this earth, we are by no means, however, forced to admit the appearance in any case of the disembodied spirit in a visible form, whether to the mental vision, during sleep, of those whom they would influence, or to their natural vision during a state of wakefulness. As efficient an influence could certainly be exercised through the similitude of dreams, excited by spiritual agency—through appropriate images awakened in the mind, or impressed upon the visual organs, or through certain chains of thought excited in the same way, as by the actual recognized presence of the spirit itself. The idea of the visible appearance of a disembodied spirit in human form, and clothed in the habiliments of every-day life, or in garments of any kind or material, is encompassed with so many difficulties, is replete with so many glaring absurdities, that a strong doubt of its correctness cannot fail to be excited whenever it is carefully and deliberately analyzed. We cannot conceive of a more incongruous image than that of an immaterial spirit clothed as a peasant or a prince, a warrior or a judge, a man or a woman of the present or of any former age.

The popular notions in respect to spiritual manifestations have always been gross and material, and they have been too implicitly adopted by historians of the supernatural. With the masses, spirits are something more than mere shadows—to them they appear as persons who had just burst from the cerements of the grave, and come forth like corpses "endowed for a brief space with semblance of life." To the unimaginative such a spiritual manifestation as that described in the Book of Job would be tame and unimpressive—"A spirit passed before my face: it stood still, but I did not discern the form thereof. An image was before my eyes, and I heard a voice." The Gælic bard never clothed the spirits of which he sang in the garments of his contemporaries, picturesque as these were, but in the clouds of the hills or in the mists of the valleys. Old Norburry, in one of his letters, describes a spirit just as it seems to us one must appear to mortal vision—if ever such a thing has or shall occur. "A form rose before me," he writes to his sister, the Abbess of an English convent, "compounded, as it were, of thin air; a flimsy yet distinct shadow, like that formed by the small fleecy cloud of summer, as it slowly floats across the azure skies, yet bearing a perfect semblance of our deceased parent as he appeared while living. No limb nor lineament could be distinctly traced, and yet the entire appearance, shadowy and unsubstantial as it was, sufficed to bring vividly before me the image of our father. It is impossible for me to describe to you of what and how the appearance was composed, and yet its totality was of a nature to suggest all that was requisite in order to convince me that the disembodied spirit of our beloved parent stood before me."

Of the appearance of disembodied spirits in a visible form to mortal eyes we have no proof; but, on the other hand, there are many reasons for doubt whether such a thing ever has occurred. Physicians are familiar with various abnormal conditions of the nervous system, attended with the most remarkable aberrations in the functions of all the external senses, in consequence of which not only are the most erroneous impressions conveyed to the mind of the objects and occurrence which actually fall within their range, but images, also, so vivid as to have all the semblance of reality, of objects and occurrences which in fact have no real existence. It is probable that there are many of the abnormal states of the nervous system which, together with the phenomena to which they give rise, have not been observed with sufficient accuracy to enable us to determine their exact nature and character. When we study attentively the phenomena of the abnormal conditions of the nervous system upon which depend double consciousness, hallucination, intense and prolonged mental abstraction, sleep-walking, ecstasy, trance, and many of the forms of delirium—conditions with which the physician is perfectly familiar, we cannot but become convinced that to aberrations in the functions, dependent upon disease of one or other of the different portions of the nervous centres and of their associated organs, are to be attributed the larger number, if not all, of the instances which have been adduced of spiritual visitations and spiritual influences, or of the action of man's immaterial nature, independently of his material organs. We know that persons in a state of ecstasy or trance, or of deep and protracted abstraction, have imagined themselves to be transported to some distant country, and there to witness occurrences that were recorded as having taken place in times gone by, or to converse with individuals of whom their sole knowledge was obtained from the report of others or from history; while others have supposed themselves to be snatched up into heaven and to have beheld its ineffable glories, in company with the souls of the just made perfect. We know, too, that many who have been the subject of these trance-dreams, on regaining their entire consciousness have recognized and admitted the unreal character of the imagery thus presented to their mental vision when in a cataleptic state. A few, it is true, have on the contrary insisted invariably upon the truthfulness—the actuality of all which they in such visions saw and heard.

It may be confidently asserted that there is not among all the narratives that have been adduced in support of ultramundane visitations or of spiritual influences and agency, with all the semblance they exhibit of thought, intention, and foresight, and even taking into account also their supposed relationship to certain subsequent events of which they would seem to be prophetic, a single one the prototype of which, more or less exact, may not be found in the phenomena exhibited by the sensual and intellectual functions when in a condition of disease.

While the foregoing considerations are sufficient to throw no little light upon the actual character of many of those occurrences which have been assumed to be of a spiritual nature by certain writers, they by no means, however, prove that man is not subject to an influence emanating from rational spiritual existences invisible to his senses.

Some years ago we read with deep interest a discourse, by an English divine of the name of Bronten, on "the future state of existence," and were most favorably impressed with the leading suggestions of the author as to the probable place and occupation of man's immortal spirit subsequent to the death of the body. It is well known that there are constantly around and about us a variety of subtile agents and forces, all of which exert upon organized and unorganized matter an influence of whose nature and full extent we are still ignorant, and by the operation of which the entire phenomena of life are controlled and modified to a far greater extent perhaps than we are yet aware. If, then, argues the author of the discourse alluded to, it be true that caloric, electricity, magnetism, gravitation, with the air amid which we move and breathe, and, it may be, many other subtile agents, of whose very existence we as yet dream not, but upon the constant operation of all of which our very being depends, may perform their wondrous offices in immediate contact with our bodies, while in their ordinary estate they escape entirely the cognizance of our senses, we cannot reject as impossible the supposition that the immaterial spirits of our departed relatives and friends may exist in an invisible and intangible state in our immediate vicinity. Nor can we deny, merely because we are unconscious of it, that these spirits exercise an influence over our thoughts and motives and impulses, that may, without absolutely controlling our actions, modify them in a manner adapted to increase our moral and physical well-being, and to an extent commensurate with our own willingness to be guided by the secret influence to which we are thus subjected. Without professing ourselves to be converts to the doctrine set forth by Mr. Bronten in the discourse referred to, we admit that it is one not devoid of probability—while it is, confessedly, replete with beauty and with consolation.

Mr. Owen insists that one advantage of the interference of the spirits of the departed in the affairs of men, is to convince us of the certainty of our continued existence after the death of the body, and to communicate to us some idea of the nature of that existence. Before we can be permitted to speculate as to the purposes to which spiritual manifestations and spiritual agency may serve, the actuality of such manifestations and agency must first be established; and perhaps we have a right to demand also the proof that a knowledge of the immortality of our souls, and of their condition when finally separated from the body, cannot be as fully acquired without, as it could be through the visitation or influence of disembodied spirits.

That man possesses an intellectual, spiritual, and immortal nature, distinct from the material organism with which, during the lifetime of the individual, it is connected, and through which its functions are performed —which spiritual portion is capable of a separate, active, and independent existence, when the body shall become disorganized and resolved into its constituent elements—are propositions most clearly deducible from revelation, while they are confirmed by all the evidence derivable from a study of the physiology of the human being in its widest bearings and ultimate results. All that we know of man, the more intimately we inquire into his nature the more certain it becomes that the mind or intellect, the knowing, comparing, judging, willing power, is a reality distinct from the body, independent as well of life as of mere organization, and that, consequently, man is to be viewed as a compound being, having, at the same time, two active, controlling absolute principles—animal life and intellectual life—"mysteriously united in an individual material frame, and running in parallel lines until the hour when death shall separate them."

What, however, is the exact nature of man's soul or spirit, his ever-enduring spiritual life—whether it consist simply of purely intellectual and moral perceptions and acts, or of a very subtile, ethereal substance, differing essentially from all that we are accustomed to recognize as matter, we have no means of determining with anything approaching to certainty. It is, nevertheless, a very probable supposition, that "the spiritual body," with which man is clothed in the resurrection, may be composed of some refined, exalted, and spiritual material, organized in a manner that shall render it incapable of change, and consequently of decay and death. That such is the case, we are perhaps warranted in assuming from the light, small, it is true, in amount, that has been revealed to us in respect to the subject, and from the seeming necessity of some such organization in order to insure the recognition and intercourse of disembodied spirits in the future world. From the so-called spiritual manifestations that have been recorded by various writers and at various times, it is very certain that we can derive no more fixed views in relation to man's future state and condition than from the sources we have indicated. The revelations said to have been made by spiritual agency of the condition of man's immortal nature, when set free from its earthly prison, are contradictory in the extreme—being nothing further, it is evident, than a reflection of the particular theological notions and metaphysical deductions of the persons by whom the supposed spiritual manifestations were observed.

Were not the argument for or against the reality of ultramundane influences or of independent spiritual agency, based upon the good or evil that may thence result to man during his present state of being, a very poor and inconclusive one, we should be inclined to show in opposition to the views advanced by Mr. Owen, and we believe it would be in our power to do so without much difficulty, that mischievous consequences of a very serious character would necessarily ensue were spirits, able and willing to perform the acts which are recorded of them, permitted to interfere with the affairs of human life.

In the foregoing imperfect and feeble review of a work replete, confessedly, with the deepest interest and no trifling power, we have not attempted to present anything having the semblance of an analysis of the several chapters in the course of which the author has gradually developed his views of spiritual phenomena and agency, nor of the very specious chain of reasoning by which he attempts to illustrate and enforce those views and to obviate the objections to which he is evidently conscious that they are amenable. Such an analysis would extend our notice of the book to a most unreasonable length. And for the same reason we have been unable to examine in detail the several narratives presented in proof and exemplification of the particular phenomena which are the subject of discussion, or to notice critically the special comments with which these narratives are accompanied.

To the correctness of many of the leading propositions advanced by Mr. Owen, we yield a willing assent, even when we are forced to deny the legitimacy of some, at least, of the important conclusions he has deduced from them. Instead of following Mr. Owen step by step throughout his inquiry, we have preferred to take up the subject of it in its more general aspect, and simply indicate what, in our opinion, constitute a few of the leading difficulties which stand in the way of our receiving as clear deductions from the facts presented, the particular doctrines to establish which is evidently the leading object of the publication before us.

One good may possibly be wrought by the work of Mr. Owen, besides furnishing us with the means by which a few spare hours may be most agreeably spent, and that is, by giving to the narratives it contains—the very best we presume that could be selected to prove the verity of spiritual phenomena—a wide circulation, and in a form and manner that will commend them not merely to the notice of the credulous, who delight in the wild and the supernatural, without stopping to inquire into its probability even, but to the serious consideration of those cool and inquiring minds who insist upon subjecting everything which demands their credence to the closest scrutiny and most searching analysis, and in this manner to provoke the eduction of additional facts and arguments in support or in refutation of the doctrines of spiritual manifestations and influences, so that the truth, on whichever side of the question it may lie, may be positively and fully established.

